# Impact of Yoga and Meditation on Cellular Aging in Apparently Healthy Individuals: A Prospective, Open-Label Single-Arm Exploratory Study

**DOI:** 10.1155/2017/7928981

**Published:** 2017-01-16

**Authors:** Madhuri Tolahunase, Rajesh Sagar, Rima Dada

**Affiliations:** ^1^Lab for Molecular Reproduction and Genetics, Department of Anatomy, All India Institute of Medical Sciences (AIIMS), New Delhi, India; ^2^Department of Psychiatry, All India Institute of Medical Sciences (AIIMS), New Delhi, India

## Abstract

This study was designed to explore the impact of Yoga and Meditation based lifestyle intervention (YMLI) on cellular aging in apparently healthy individuals. During this 12-week prospective, open-label, single arm exploratory study, 96 apparently healthy individuals were enrolled to receive YMLI. The primary endpoints were assessment of the change in levels of cardinal biomarkers of cellular aging in blood from baseline to week 12, which included DNA damage marker 8-hydroxy-2′-deoxyguanosine (8-OH2dG), oxidative stress markers reactive oxygen species (ROS), and total antioxidant capacity (TAC), and telomere attrition markers telomere length and telomerase activity. The secondary endpoints were assessment of metabotrophic blood biomarkers associated with cellular aging, which included cortisol, *β*-endorphin, IL-6, BDNF, and sirtuin-1. After 12 weeks of YMLI, there were significant improvements in both the cardinal biomarkers of cellular aging and the metabotrophic biomarkers influencing cellular aging compared to baseline values. The mean levels of 8-OH2dG, ROS, cortisol, and IL-6 were significantly lower and mean levels of TAC, telomerase activity, *β*-endorphin, BDNF, and sirtuin-1 were significantly increased (all values *p* < 0.05) post-YMLI. The mean level of telomere length was increased but the finding was not significant (*p* = 0.069). YMLI significantly reduced the rate of cellular aging in apparently healthy population.

## 1. Introduction

In the last decade there has been a significant increase in complex lifestyle diseases like depression, diabetes mellitus (DM), cardiovascular diseases (CVD), cancer, and infertility. These diseases are strongly associated with accelerated cellular aging [1, 2] and have become the bane of modern society [3–5]. Within a homogeneous sample of apparently healthy adult population, biomarkers have been defined recently [6] to characterize the complex processes of accelerated aging phenomenon. Although we do not have any gold standard biomarker to monitor healthy aging, based on the current knowledge of putative biomarkers, the cardinal biomarkers of cellular aging and metabotophic biomarkers which can influence them have become the focus of latest translational research to develop interventions to prevent chronic lifestyle diseases.

The cardinal biomarkers of cellular aging include DNA damage, telomere length attrition, and oxidative stress (OS) [7].* DNA damage* causes genomic instability which is responsible for cellular dysfunctions in the pathogenesis of lifestyle diseases [8–10]. OS is the most important cause for DNA damage. Although many different oxidative DNA damage (ODD) products have been identified 8-OH2dG (8-hydroxy-2′-deoxyguanosine), a highly mutagenic oxidative DNA adduct has been the subject of intensive study and is a definitive biomarker of DNA damage [11].* Telomere attrition* is due to altered telomere metabolism involving decrease in telomerase enzyme activity and OS. It contributes to genomic instability and is associated with aging and lifestyle diseases [12].

*Oxidative stress*, an imbalance between the prooxidants and the antioxidant defense mechanisms, becomes pathological at both extremes of the physiological range needed for normal cellular functions. It is involved in the pathogenesis of complex lifestyle and chronic diseases [13] including depression [14], obesity [15], and infertility [16, 17], the leading public health problems.

Several metabotrophic blood biomarkers influencing cellular aging include biomarkers of stress and inflammatory response, neuroplasticity, and longevity.* Sustained stress response* due to chronic stress stimuli causes constantly increased cortisol levels [18], which lead to systemic tissue abnormalities like increased adiposity and neurodegeneration. The level of stress responsiveness (cortisol levels) can be a biomarker for predicting susceptibility to lifestyle diseases [19]. Accelerated aging is characterized by a chronic, low-grade inflammation* (“inflammaging”)*. Inflammaging is a highly significant risk factor for most of chronic lifestyle diseases [20] and is a potential modifiable target [21]. IL-6 is the most prominent cytokine in inflammaging and is both a marker of inflammatory status and a hallmark of chronic morbidity [22]. Impaired* neuroplasticity* due to accelerated aging can have negative influence across the entire lifespan [23]. BDNF is a major regulator of neuroplasticity [24], which may be increased in specific regions of the brain by various interventions [25].* Health span and longevity* are influenced by several factors. Sirtuin-1 (SIRT1), a histone deacetylase (HDAC), is prominent among them and recently has become a target for various interventions [26]. It systemically influences nutrition and energy metabolism and centrally has a role in circadian rhythm, survival against stress [27] and neuronal plasticity [28].

A variety of interventions have been studied [29, 30] to determine their influence on preventing lifestyle diseases and promoting health and longevity. They include drugs targeting specific hallmarks of aging, namely, physical exercise [31], nutrition, caloric restriction [32], and antioxidants [33]. However, no single intervention is shown to be an effective preventive and therapeutic strategy for modern complex lifestyle diseases and provide comprehensive benefits for delaying or reversing accelerated aging. Therefore, further research is needed to find optimum interventions for population at risk of lifestyle diseases. Yoga is an emerging integrative health discipline, which can positively modulate mind and body [34] and has been shown to improve clinical profile of patients with various pathologies [35] including depression, obesity, hypertension, asthma, type II diabetes, and cancer. However, recent reviews on Yoga suggest that potential underlying mechanisms need to be further explored [36]. Studies on biomarkers of disease and health in Yoga based interventions are limited and they have only highlighted diabetic and lipid profiles [37, 38], stress and inflammatory markers [39, 40] and neuroimaging correlates [41], in populations with specific medical conditions. Evidence is lacking regarding the efficacy of Yoga lasting short duration of 3 to 12 weeks in improving the biomarkers of cellular aging in apparently healthy people. Thus, the present study was designed to evaluate the impact of Yoga and Meditation based lifestyle intervention (YMLI) on cellular aging and longevity by analyzing cardinal and metabotrophic biomarkers in the peripheral blood of apparently healthy subjects.

## 2. Materials and Methods

### 2.1. Study Design and Participants

Ninety-six apparently healthy people were enrolled in this 12-week prospective, open-label, single arm exploratory study, from Aug 2015 to May 2016, designed to explore the impact of YMLI on cellular aging. The key inclusion criteria were male or female aged 30–65 years and leading unhealthy modern lifestyle. The key exclusion criteria were inability to perform the yogic exercises due to any physical challenges and those with recent changes in lifestyle during last 3 months. The study was initiated after ethical clearance (ESC/T-370/22-07-2015) and the registration of the trial with Clinical Trial Registry of India (CTRI REF/2014/09/007532).

### 2.2. Procedure

#### 2.2.1. Yoga and Meditation Based Lifestyle Intervention (YMLI)

Eligible subjects were enrolled in the study after screening and baseline characteristics were recorded. Participants underwent 12-week pretested YMLI program comprising theory and practice sessions [42, 43]. YMLI is designed to be an integrative health strategy incorporating the classic components of Yoga including Asanas (physical postures), Pranayama (breathing exercises), and Dhayna (Meditation) which are derived from a mix of Hatha Yoga and Raja Yoga. The YMLI for the current study was suitably modified for apparently healthy subjects. YMLI program included sessions 5 days per week for 12 wks. For the first two weeks the sessions were held at integrated health clinic (IHC), AIIMS, New Delhi, and taught by registered, specialized Yoga instructors (educational qualifications include Bachelor of Naturopathy and Yoga Sciences and P.G. Diploma in Yoga Therapy). Remaining 10 weeks were home based. Monitoring of compliance of the home based YMLI was through maintenance of a dairy and telephonic contact. The details of the activities in a day during YMLI program are given in Table 1. Each session in YMLI included a set of Asanas (physical postures), Pranayama (breathing exercises), and Dhayna (Meditation) for approximately 90 minutes. This was followed by an interactive lecture (only during the first two weeks of YMLI at IHC) on lifestyle, lifestyle diseases, and importance of their prevention for 30 minutes.

#### 2.2.2. Laboratory Procedures

During this 12-week study the participants were evaluated for various biomarkers on day 0 and week 12. Fasting venous blood samples (5 mL) were collected and divided into two parts. One part was allowed to clot and the serum was separated within 30 min and the other part was transferred to heparinized/EDTA vials and was centrifuged at 2000*g* for 15 minutes at 4°C. Both serum and plasma were stored at −80°C until analyzed. ROS detection was done by chemiluminescence assay (Berthold detection luminometer, USA). Peripheral blood leukocyte telomere length was measured by qPCR method and telomerase activity was determined by using a telomerase assay kit (Roche, Switzerland), as per manufacturer's protocol. 8-OH2dG was estimated in white blood cell DNA (Cayman's EIA kit). ELISA kits were used for levels of TAC (Cayman Chemical, Ann Arbor, USA), cortisol (DRG Diagnostic, Germany), *β*-endorphin (Phoenix Pharmaceuticals, Inc.), IL-6 (Gen-Probe, Diaclone Diagnostic, France), BDNF (Raybiotech, Inc), and sirtuin-1 (Quayee Bio-Technology). Quality-control assays for biomarkers and validation were performed.

#### 2.2.3. Endpoints

The primary endpoint was to assess the change in levels of cardinal biomarkers of cellular aging from baseline to week 12. The biomarkers included the following: 8-OH2dG, ROS, and TAC (markers of OS and ODD) and telomere attrition markers telomere length and telomerase activity. The secondary endpoints were assessment of metabotrophic blood biomarkers associated with cellular aging, which included cortisol, *β*-endorphin, IL-6, BDNF, and sirtuin-1 from baseline to week 12.

### 2.3. Statistical Analysis

Data were analyzed using SPSS 20 (IBM Corp, Armonk, NY). Descriptive statistics are reported as means and standard deviations. Changes in outcome variables were analyzed using paired-samples* t-*test. Exploratory analysis included comparisons for within gender subgroups using paired-sample* t*-test. Significance was accepted at *p* < 0.05.

## 3. Results

**Figure 1:**
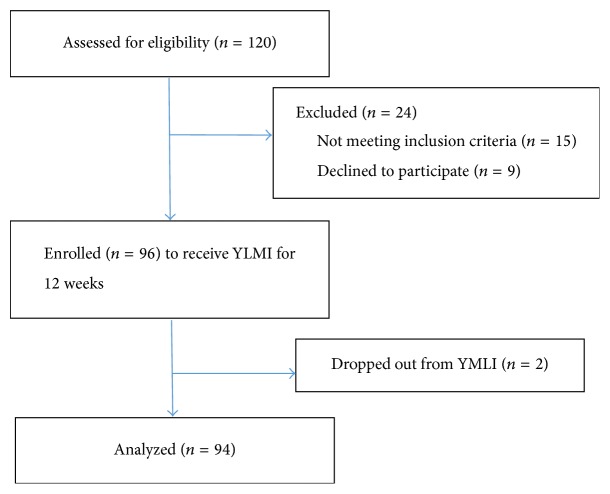
Flow diagram of study participation.

The flow diagram of participation details is provided in Figure 1. Of 96 subjects, 94 subjects were assessed for impact analysis. Two subjects were excluded from analysis due to poor compliance to the program. Baseline sociodemographic characteristics are shown in Table 2.

After 12 weeks of YMLI, there was significant improvement in both cardinal and metabotrophic biomarkers of cellular aging compared to baseline values (Table 3). The mean levels of 8-OH2dG and ROS were significantly lower and mean levels of TAC and telomerase activity were significantly increased (all values *p* < 0.05). The mean level of telomere length was increased but finding was not significant (*p* = 0.069). The mean levels of cortisol and IL-6 were significantly lower and mean levels of *β*-endorphin, BDNF, and sirtuin-1 were significantly increased (all values *p* < 0.05).

Few differences were noted in the gender subgroup analysis. Only male subgroup showed significant decrease in the levels of IL-6 and a more marked reduction in cortisol levels (males *p* = 0.001; females *p* = 0.036). After 12 weeks of YMLI, we also noted significantly reduced BMI in the study population (*p* < 0.01).

## 4. Discussion

The results of this study highlight the positive impact of YMLI on biomarkers of cellular aging and in promoting cellular longevity through changes in both cardinal and metabotrophic biomarkers. The findings suggest that the impact is mediated through improvement in genomic stability, telomere metabolism, and balance of cellular oxidative stress, well-regulated stress and inflammatory responses, and increase in neuroplasticity and nutrition sensing.

Genomic stability is central to cellular longevity and disease-free youthful healthy life and findings from our study suggest the reduction of genomic instability (decreased levels of 8-OH2dG) by YMLI. Unhealthy social habits (smoking, excess alcohol intake, etc.), sedentary lifestyle, exposure to environmental pollutants, and intake of processed and nutritionally depleted food have taken a toll on human health with onset of lifestyle diseases at a much younger age [3–5]. These environmental and lifestyle factors are responsible for genomic instability [10]. DNA damage to both mitochondrial and nuclear genome from endogenous as well as exogenous insults results in accumulation of genetic aberrations and genome hypermutability [8–10].

This is mainly due to aberrant DNA damage response (DDR) pathway, which is essential for DNA repair and for monitoring genomic integrity. Deficient DNA repair triggers systemic effects to promote pathological aging [10]. Reduction of DNA damage by YMLI suggests potential of yoga in activating DDR pathway to repair genomic damage and improve genomic stability and changes in metabotrophic factors seen in the study may be associated with these benefits.

Maintaining telomere length through regulation of telomere metabolism contributes to genomic stability and reduction in telomere attrition (increase in telomere length and telomerase activity levels) shown by our study after YMLI suggests the potential for yoga in telomere metabolism and cellular longevity. Telomeres, which serve as a biological clock, are highly conserved hexameric repeats and maintaining their length is vital for cellular longevity. Telomerase is an important regulator of telomere length and accurate regulation of its activity, and a correct telomere-telomerase interaction is important to precisely safeguard telomere length and prevent telomere attrition [44]. ODD is prominent among the factors which can adversely affect telomere length [45]. Rapid telomere attrition due to ODD is associated with senescence and related disease conditions [46, 47]. Improved telomere metabolism after YMLI seen in the study may contribute to genomic stability. More research is needed to explore the mechanisms of how yoga and meditation intervention can positively modify telomere metabolism.

Our study suggests that improvement in maintenance of balance in cellular oxidative stress (decrease in ROS and increase in TAC) by YMLI. Supraphysiological ROS levels are due to endogenous and exogenous factors like smoking, excess alcohol consumption, exposure to electromagnetic radiation, infection, xenobiotic exposure, and psychological stress [48]. Even the levels of ROS below physiological limits are deleterious to normal cellular function and maintaining OS at physiological levels is important for cellular longevity. Increased OS causes damage to all molecules, including damage to DNA and telomeres. It also affects signal transduction and gene transcription by causing genome wide hypomethylation [49] and thus causes changes in the epigenome. Regulation of cellular oxidative stress within physiological limits after YMLI suggests the potential of this intervention in protecting cells from OS induced DNA damage and telomere attrition and in reversing epigenetic changes, which are accumulated due to unhealthy lifestyle and adverse environmental conditions. Other studies [50] support these findings and have shown reduced OS upregulation of telomerase activity and decreased ODD after YMLI. To combat OS people use antioxidants without monitoring ROS levels resulting in reductive stress [51] unlike in YMLI which regulates ROS levels so that no redox sensitive physiological functions are impaired.

Modern lifestyle and associated psychological stress have complex interactions with lifestyle habits, environmental conditions, and medical interventions to cause accelerated cellular aging, which adversely affect our mental, physical, and reproductive fitness [3–5]. Improved cellular longevity after YMLI suggests the potential role of Yoga in promoting this fitness. While psychological stress is a major manifestation on mind contributing to increased prevalence of neuropsychiatric disorders including depression, abnormal fat accumulation is a major somatic manifestation contributing to increased prevalence of metabolic syndrome and all the diseases that come under the umbrella of metabolic syndrome including, obesity, DM, and CVD [52]. Other peripheral manifestations of unhealthy modern lifestyle include aging of gonads leading to infertility [53] and recurrent pregnancy loss. Previous studies have demonstrated the clinical benefits of Yoga and Meditation in all these medical conditions [35]. Dada et al. have shown that YMLI can reduce testicular aging and result in significant upregulation in telomerase activity and decline in seminal OS and ODD [8]. The ongoing studies in our laboratory on the impact of Yoga and Meditation have provided significant evidence for the reversal of cellular aging in subjects prone to accelerated aging due to depression. Microarray (Agilent 8 × 60k Microarray kit) and analysis of gene expression pre- and post-YMLI showed decreased IL6, IL10, and MAP10 and increased IL2 and IL4 [8, 54, 55]. Improved cellular longevity seen in our study after YMLI suggests that changes in both cardinal and metabotrophic biomarkers of cellular aging may be a mechanism for preventing chronic lifestyle diseases. Our study suggests that the changes in metabotrophic factors, which include increase in levels of *β*-endorphin, BDNF, and sirtuin-1 and decrease in levels of cortisol and IL-6, and the cellular processes involving them, may have important roles in reversal of cellular aging and improving cellular longevity after YMLI.

Improvement in stress and inflammatory response in our study after YMLI may be mediated by changes in cortisol, *β*-endorphin, IL-6, and other factors, with regulation by changes in brain through hypothalamic-pituitary-adrenal (HPA) axis. The response may involve regulation of adaptive pathways including integrated stress response (ISR) [18], which activate the eukaryotic translation initiation factor 2 alpha (eIF2*α*), that promote cellular recovery driving the signaling toward cell survival and longevity. The response may lead to decreased OS and reversal of senescent secretory phenotype of cells, including cells in brain, adipose tissue, endothelium, and gonads. Changes in secretory phenotype include decreased IL-6 [56], increased BDNF, and sirtuin-1 [57]. These regulated factors may lead to balance in OS and cellular longevity and contribute to tissue revival throughout the body from neuroplasticity in CNS to gonads, vessels, and muscles in the periphery. Secretory phenotype from somatic cells provides regulatory feedback to brain [58], which completes the vicious cycle of regulation between mind and body. Neurodegeneration is associated with pathogenesis of several neuropsychiatric conditions and neuroplasticity has a central role in their management and for vitality. Increased BDNF, sirtuin-1, and *β*-endorphin and decreased cortisol, which decrease cellular aging in brain, decrease neurodegeneration and increase neuroplasticity [23]. Increased cellular longevity and increased neuroplasticity may be a mechanism for alteration of gray matter volume in different regions of the cerebral cortex [59], increased mindfulness [60], and several other complex processes [55, 61] involved in reduction of stress and depression after Yoga and Meditation. Regulated mind-body communications may lead to minimization of subclinical inflammation and activation of nutrition and energy sensing pathways promoting longevity, where decrease in IL-6 and increase in sirtuin-1 play a prominent role, respectively. Previous studies have demonstrated increase in sirtuin-1 levels after interventions with caloric restriction [62]. Our study is the first to document increase in sirtuin-1 levels independent of caloric restriction after practicing Yoga. These improved processes may result in delaying onset and slowing down progression of diseases associated with accelerated cellular aging.

The impact of the intervention in both genders was assessed separately since men and women respond differently to day to day stress [63]. Interestingly, the gender subgroup analysis showed that reduction in cortisol and IL6 levels were more pronounced in male than in female subjects. No significant gender differences were seen in other biomarkers. Phase of the menstrual cycle should be taken into consideration since some biomarker levels are known to vary with different phases of the menstrual cycle [64, 65]. Our study showed significant decrease in BMI in apparently healthy subjects which came into normal range (23.64 ± 3.55) from baseline overweight range (26.30 ± 3.40). While latest research [66] suggests people with mean BMI of 27, who are overweight by current classification of obesity, are likely to survive longest in western population, similar data is not available for Indian population. Therefore, our findings need to be interpreted cautiously.

Stratification of cases was not done in this study to do subgroup analysis due to small sample size.

Lifestyle is an integrated entity, and an intervention, like YMLI, that has overall positive influence on our health appears most useful versus changing only one aspect at a time, as is seen by action of certain drugs. Yoga is holistic and a mind-body medicine and is more beneficial and advantageous than individual interventions like physical exercise, caloric restriction, and antioxidants. The practice of Yoga and physical exercise are different entities, the former results in energy conservation with economy of energy expenditure for mental and physical benefits, and the later results in energy expenditure more for physical exertions and metabolic needs, as is evident from a study which showed exercise causes erratic changes in biomarkers and results in OS [67] while Meditation brings about uniform biomarker and behavioral changes and improvement in cognition and decrease OS [68]. Therapeutic antioxidants can only decrease ROS rather than regulating it and may paradoxically shorten life span [69] due to imbalance in ROS mediated immune response [70]. YMLI regulates ROS rather than simply lowering them by balanced stress-related processes and appropriate gene expressions [71]. The only limitation of our study is that it is a single arm proof of concept study and did not include controls. It is important to adopt a lifestyle which slows the decline in health by reversing or delaying accelerated aging due to unhealthy lifestyle. The biomarkers of cellular aging can form the basis for determining the risk of chronic lifestyle diseases and the efficacy and usefulness of interventions to decrease disease risk. Hence, findings from this study are supportive of YMLI as a significant clinical utility especially in prevention of and management of complex multifactorial diseases and reducing the rate of functional decline with aging.

## 5. Conclusion

Though we cannot change our biology or chronological age we can definitely reverse/slow down the pace at which we age by adopting YMLI. This is the first study to demonstrate improvement in both cardinal and metabotrophic biomarkers of cellular aging and longevity in apparently healthy population after Yoga and Meditation based lifestyle intervention. So our health and the rate at which we age entirely depends on our choices. Making Yoga and Meditation an integral part of our lifestyle may hold the key to delay aging or aging gracefully, prevent onset of multifactorial complex lifestyle diseases, promote mental, physical, and reproductive health, and prolong youthful healthy life.

## Figures and Tables

**Table 1:** Details of activities in a day of Yoga and Meditation based Lifestyle Intervention (YMLI) program.

S. No.	Practice to be done			Duration
(1)	Session preparation instructions			5 min

(2)	Prayer			3 min
	Loosening practices (warm-up)			5 min

(3)	Asanas (Postures)	Supine	Shavasana	2 min
			Uttanpadasana	2 min
			Pawanmuktasana	2 min
		Prone	Makarasana	2 min
			Bhujangasana	2 min
			Salabhasana	2 min
		Sitting	Vakrasana	2 min
			Ardha-Matsyendrasana	2 min
			Vajrasana	2 min
		Standing	Tadasana	2 min
			Vrikshasana	2 min
			Ardhachakrasana	2 min

(4)	Relaxation		Shavasana	5 min

(5)	Pranayama (Breathing Exercises)		Nadishodhana	20 min
			Bhramri	
			Shitkari	
			Shitali	
			Brahmamudra	

(6)	Aumkar recitation			3 min

(7)	Dhyana (Meditation)			20 min

(8)	Shanti mantra			5 min

(9)	Interactive session (first 2 weeks only at Integrated Health Clinic, AIIMS, New Delhi)			30 min

Total				120 min

**Table 2:** Sociodemographic characteristics of participants.

Variable	Values
Age (years)	**40.26 **(**10.13**)
Sex	
Female	**52 **(**55.32**)
Male	**42 **(**44.68**)
Socioeconomic status	
Kuppuswamy socioeconomic status scale	
Education	4.82 (1.24)
Occupation	5.26 (2.38)
Income	8.60 (2.74)
*Total*	**18.68 **(**7.34**)
BMI (kg/m^2^)	**26.30 **(**3.40**)

Data were described as frequency (%) for sex and mean (SD) for others.

**Table 3:** Change in outcomes in apparently healthy sedentary subjects participating in a Yoga and Meditation based lifestyle intervention (*n* = 94).

Characteristics	Baseline	12 wks	Change from baseline to 12 wks (diff. 95% C.I.)	Effect size^*∗*^	*p* value
Primary endpoints: cardinal biomarkers of cellular aging					
*Oxidative stress*					
ROS (RLU/min/10^4^ neutrophils)	1215.069 ± 88	1020.81 ± 79	194.3 (164, 224.5)	0.7	<0.0001
TAC (mmol Trolox equiv/L)	5.94 ± 1.52	7.4 ± 2.1	−1.16 (−1.9, −0.41)	0.4	<0.001
*DNA damage*					
8OH2dG (pg/mL)	1026.23 ± 630	790.98 ± 400	235.3 (72.73, 397.8)	0.22	<0.01
*Telomere attrition*					
Telomerase activity (IU/cell)	1.89 ± 1.42	2.94 ± 2.2	−1.05 (−1.68, −0.41)	0.3	<0.001
Telomere length (IU/cell)	2.36 ± 1.6	2.44 ± 1.4	−0.08 (−0.61, 0.45)	0.02	0.069
Secondary endpoints: biomarkers associated with cellular aging					
Cortisol (ng/mL)	118.83 ± 50.50	96.32 ± 38.6	22.51 (7.6, 37.42)	0.3	<0.01
Interleukin (IL6) (pg/mL)	3.16 ± 2.42	1.94 ± 2.3	1.22 (0.47, 1.97)	0.3	<0.001
*β*-Endorphins (ng/mL)	6.2 ± 3.5	8.2 ± 4.2	−2 (−3.22, −0.77)	0.3	<0.001
BDNF (ng/mL)	19.7 ± 6.75	37.1 ± 5.6	−17.4 (−19.48, −15.32)	0.7	<0.0001
Sirtuin (ng/mL)	26.69 ± 10.42	40.64 ± 11.6	−13.95 (−23.41, −4.49)	0.5	<0.01
*BMI (kg/m* ^*2*^)	26.30 ± 3.40	23.64 ± 3.55	2.66 (0.56, 3.12)	0.4	<0.01

^*∗*^Effect size was calculated by dividing change by standard deviation at baseline of the specific outcome and interpreted using Cohen's *d* (small effect: 0.2 to 0.3, medium effect: 0.5, and large effect: 0.8).
